# The Role of High-Resolution Magic Angle Spinning 1H Nuclear Magnetic Resonance Spectroscopy for Predicting the Invasive Component in Patients with Ductal Carcinoma In Situ Diagnosed on Preoperative Biopsy

**DOI:** 10.1371/journal.pone.0161038

**Published:** 2016-08-25

**Authors:** Eun Young Chae, Hee Jung Shin, Suhkmann Kim, Hyeon-Man Baek, Dahye Yoon, Siwon Kim, Ye Eun Shim, Hak Hee Kim, Joo Hee Cha, Woo Jung Choi, Jeong Hyun Lee, Ji Hoon Shin, Hee Jin Lee, Gyungyub Gong

**Affiliations:** 1 Department of Radiology and Research Institute of Radiology, Asan Medical Center, University of Ulsan College of Medicine, Seoul, South Korea; 2 Department of Chemistry and Chemistry Institute for Functional Materials, Pusan National University, Busan, South Korea; 3 Center for Magnetic Resonance Research, Korea Basic Science Institute, Chungbuk, South Korea; 4 Department of Bio-Analytical Science, Korea University of Science and Technology, Daejeon, South Korea; 5 University of Ulsan, College of Medicine, Seoul, South Korea; 6 Department of Pathology, Asan Medical Center, University of Ulsan College of Medicine, Seoul, South Korea; Linköping University, SWEDEN

## Abstract

The purpose of this study was to evaluate the role of high-resolution magic angle spinning (HR-MAS) 1H nuclear magnetic resonance (NMR) spectroscopy in patients with ductal carcinoma *in situ* (DCIS) diagnosed on preoperative biopsy. We investigated whether the metabolic profiling of tissue samples using HR-MAS 1H NMR spectroscopy could be used to distinguish between DCIS lesions with or without an invasive component. Our institutional review board approved this combined retrospective and prospective study. Tissue samples were collected from 30 patients with pure DCIS and from 30 with DCIS accompanying invasive carcinoma. All patients were diagnosed with DCIS by preoperative core-needle biopsy and underwent surgical resection. The metabolic profiling of tissue samples was performed by HR-MAS 1H NMR spectroscopy. All observable metabolite signals were identified and quantified in all tissue samples. Metabolite intensity normalized by total spectral intensities was compared according to the tumor type using the Mann-Whitney test. Multivariate analysis was performed with orthogonal projections to latent structure-discriminant analysis (OPLS-DA). By univariate analysis, the metabolite concentrations of choline-containing compounds obtained with HR-MAS 1H NMR spectroscopy did not differ significantly between the pure DCIS and DCIS accompanying invasive carcinoma groups. However, the GPC/PC ratio was higher in the pure DCIS group than in the DCIS accompanying invasive carcinoma group (p = 0.004, Bonferroni-corrected p = 0.064), as well as the concentration of myo-inositol and succinate. By multivariate analysis, the OPLS-DA models built with HR-MAS MR metabolic profiles could clearly discriminate between pure DCIS and DCIS accompanying invasive carcinoma. Our preliminary results suggest that HR-MAS MR metabolomics on breast tissue may be able to distinguish between DCIS lesions with or without an invasive component.

## Introduction

Although the diagnosis of ductal carcinoma *in situ (*DCIS) is now more common with the more widespread use of mammographic screening [1], there is substantially imperfect understanding of the biology and clinical outcome of DCIS [2]. DCIS of the breast consists of proliferating malignant cells within the lumen of the mammary duct and with no evidence of invasion beyond the basement membrane [3]. It is estimated that 14% to 50% of DCIS lesions progress to invasive breast cancer if left untreated [4]. Because some DCIS lesions rapidly progress to invasive carcinoma, whereas others remain indolent, distinguishing between the two remains a primary challenge regarding the optimal selection of treatment.

*Ex vivo* high-resolution magic angle spinning (HR-MAS) 1H nuclear magnetic resonance (NMR) spectroscopy provides highly resolved spectra from intact biological tissue. HR-MAS analysis of tissue samples provides peaks representative of metabolites [5]. This method provides comprehensive and detailed information on the biochemical composition of the tissue and can be used in non-targeted analysis to identify surrogate markers predicting malignant transformation or treatment response. Accordingly, HR-MAS MR spectroscopy has been used in breast cancer studies, for metabolite identification, diagnosis, characterization, and correlation with prognostic markers of breast cancer and treatment response monitoring [5–8]. However, these studies mainly focused on the analysis of invasive breast cancer and little is known regarding the use of HR-MAS to evaluate DCIS of the breast.

The purpose of our current study was to evaluate the role of HR-MAS 1H NMR spectroscopy in patients with DCIS diagnosed on preoperative biopsy. We investigated whether the metabolic profiling of tissue samples using HR-MAS 1H NMR spectroscopy would help to distinguish between DCIS lesions with or without an invasive component.

## Materials and Methods

### Patient Selection

The institutional review board of Asan Medical Center approved this combined retrospective and prospective study (2013–1126). Patient records or information was anonymized and de-identified prior to analysis. Inclusion in this study was based on the following criteria: (1) a diagnosis of DCIS obtained by preoperative core-needle biopsy; (2) had undergone surgical treatment; and (3) had a final diagnosis of pure DCIS or DCIS accompanying invasive carcinoma. Using sample size calculation, a total sample size of 56 (28 study subjects in each group) would achieve 86% power to detect a change in sensitivity for predicting the invasive component in patients diagnosed as DCIS from 0.7 to 0.9 and in specificity from 0.7 to 0.9 using a one-sided binomial test. Between April 2007 and June 2013, 48 patients who fulfilled the criteria were identified from the Bio-Resource Center at the Asan Medical Center Korea Biobank Network (18 patients with pure DCIS and 30 with DCIS accompanying invasive carcinoma). For these patients, informed consent was substituted by previous agreement obtained before registration with the Bio-Resource Center. For the prospective part of the study, 12 consecutive patients with pure DCIS were included between January and May 2014 after providing written informed consent. The total cohort comprised 30 patients with pure DCIS (age range, 43–71 years; mean, 54.0 years) and 30 with DCIS accompanying invasive carcinoma (age range, 34–73 years; mean, 48.7 years).

### Sample Preparation

All tissue samples were dissected from specimens immediately after surgery. We obtained two mirror tissues during tissue harvesting. One was used for HR-MAS 1H NMR spectroscopy and the other for the final histopathologic diagnosis. The tissue samples were primarily obtained from the DCIS component. However, they may have included some of the invasive component because the dissection was based on the gross inspection of samples. The tissue samples were placed in a cryogenic vial. During the acquisition of the tumor samples, the pathologist meticulously attempted to avoid including non-tumorous breast parenchyma, based on the macroscopic findings. The samples were snap-frozen in liquid nitrogen immediately and then stored at −70°C.

### HR-MAS 1H NMR Spectroscopy

HR-MAS 1H NMR spectroscopy was performed on the frozen tissue specimens using an NMR spectrometer (400 MHz Varian Unity-Inova; Varian Inc., Palo Alto, CA) operating at a proton NMR frequency of 400.266 MHz. All instruments were equipped with a gHX nanoprobe with a spin rate of 2000 Hz and regulated temperature at 299.1 K. While determining the machine settings, we performed the experiment at low (i.e., less than 26°C) and room temperatures. At low temperature, the resolution was not good and had elements that were difficult to measure, such as due to the freezing of water contained in the sample. Therefore, the temperature was set to 26°C after calibration with methanol. Because the experiments in all samples were performed under the same conditions, we regarded the setting as appropriate to investigate the difference in the metabolites in the two groups. The total examination time of each sample was approximately 1 h, including the scan time (about 19 min 39 s). During the total examination time, the sampling processes before the measurement were conducted in a frozen state, and the samples were exposed to 26°C only during the measurement time (about 19 min 39 s).

Frozen samples were thawed in the NMR laboratory, weighed, and placed in a HR-MAS nanoprobe® (Agilent, Walnut Creek, CA). The total volume of the sample cell was 40 μl, and tissue samples weighing an average of 25 mg were placed in the cell with the remaining volume filled with D_2_O containing 0.01% trimethylsilyl propionic acid (TSP). An inverse-detection probe equipped with a single Z gradient coil was used. The tissue samples were analyzed using a CPMG (Carr-Purcell-Meiboom-Gill) pulse sequence to impose a T2 filter. The CPMG pulse sequence was used with 5.0 µs of a 90° pulse and the total spin-spin relaxation delay (2τn) was 61.6 ms (τ = 385.0 us, n = 80). Because the macromolecules have a shorter T2 relaxation time than the metabolites (small molecules), the macromolecule signal can be excluded by adjusting the T2 delay. All data were collected at a spinning rate of 2 kHz. The spectral acquisition parameters were as follows: 9596 complex data points; 4803.1 Hz sweep width; 2 s acquisition time; 1.0 s relaxation delay; 1.5 s pre-saturation time; 1.0 ms inter-pulse delay; 256 transients; receiver auto gain; and a total acquisition time of 19 min 39 s. For the metabolite quantification, adequately long TR and short TE were used in order to neglect the T1 and T2 relaxation time difference among metabolites. The spectra were processed and analyzed using ACD software (Advanced Chemistry Development, Toronto, Ontario, Canada). Post-processing consisted of Fourier transformation, phasing, and baseline correction. Chemical shifts were referenced in relation to the creatine (Cr) signal at 3.04 ppm. Spectral regions from 1.47 to 3.60 ppm [alanine (Ala), Cr, free choline (Cho), phosphocholine (PC), glycerophosphocholine (GPC), myo-inositol (m-Ins), taurine (Tau), glycine (Gly), and succinate (Suc)] were selected for quantification. The glycerol signal was assigned using the Chenomx Reference Compound library. The peak amplitudes of the metabolites were measured by fitting a Voigt (e.g., Gauss-Lorentz) line-shape function. The integration values were normalized according to the number of contributing protons per molecule and to tissue weight. Quantification was performed by comparing the integrated TSP signal (2 mM in each sample) to the signal of interest in the tumor spectrum. Absolute concentrations were recorded as mM. The average volume of tissue sample was 25 mg.

### Data and Statistical Analysis

Clinicopathologic data of the included patients were collected from a review of the medical records. Associations between the two groups and the categorical variables were assessed using chi-square analysis. Exact tests were used to assess data with low cell frequencies. The continuous variable (i.e. patient age) was assessed using the t-test. Spectral data acquired by HR-MAS 1H NMR spectroscopy were expressed with metabolite concentrations [Ala, Cho, PC, GPC, total choline (tCho, the sum of Cho, PC, and GPC), Cr, m-Ins, Tau, Gly, Suc] and metabolic ratios (Cho/Cr, PC/Cr, GPC/Cr, GPC/PC, GPC/Cho, PC/Cho). Patients were grouped as pure DCIS or DCIS accompanying invasive carcinoma. On the Kolmogorov-Smirnov test for normality, the result was statistically significant (p<0.05). Therefore, HR-MAS 1H NMR spectroscopic values were compared between the two groups using the Mann-Whitney test because the data did not have a normal distribution. The Bonferroni correction was applied to address the problem of multiple comparisons and was calculated as the each p value multiplied by the number of comparisons. Then Bonferroni-corrected P value less than 0.05 was considered significant. Statistical analysis was performed with SAS for Windows, version 9.0 (SAS Institute, Cary, NC). Statistical significance was defined as a p value less than 0.05.

For multivariate analysis, ^1^H NMR spectra were binned using Chenomx NMR Suite 7.1 software (Spectral database; Edmonton, Alberta, Canada). The spectra were normalized to the total area. The spectral region between δ 0.5 and 10 ppm was divided into bins of 0.01-ppm width. The water region from 4.6 ppm to 4.9 ppm and the ethanol peaks (1.15–1.20, 3.6–3.7 ppm) were excluded prior to the analysis. The binning data were aligned using the icoshift algorithm in MATLAB (MathWorks, Natick, MA), and the data were converted to the format of SIMCA-P 11.0 (Umetrics, Sweden) in Excel (Microsoft, Seattle, WA). Pareto scaling was used to preprocess the data. Orthogonal projections to latent structure-discriminant analysis (OPLS-DA) was subsequently performed to differentiate the pure DCIS and DCIS accompanying invasive carcinoma groups. Class discrimination models were built until the cross-validated predictability value did not significantly increase so as to avoid over-fitting of the statistical model. The model was validated by prediction of unknown samples by assigning every Nth observation to the same group. With seven cross-validation groups, the first cross-validation group held observations number 7, 14, 21, and so on. The second cross-validation group held observations 1, 8, 15, and so on, and the third held observations 2, 9, 16, and so on. An *a priori* cut-off value of 0.5 was used to evaluate the prediction results [9]. Signals contributing to group discrimination were identified by an S-plot, and the corresponding HR-MAS MR spectral data were identified using Chenomx software.

## Results

### Histopathologic Analysis

**Fig 1:**
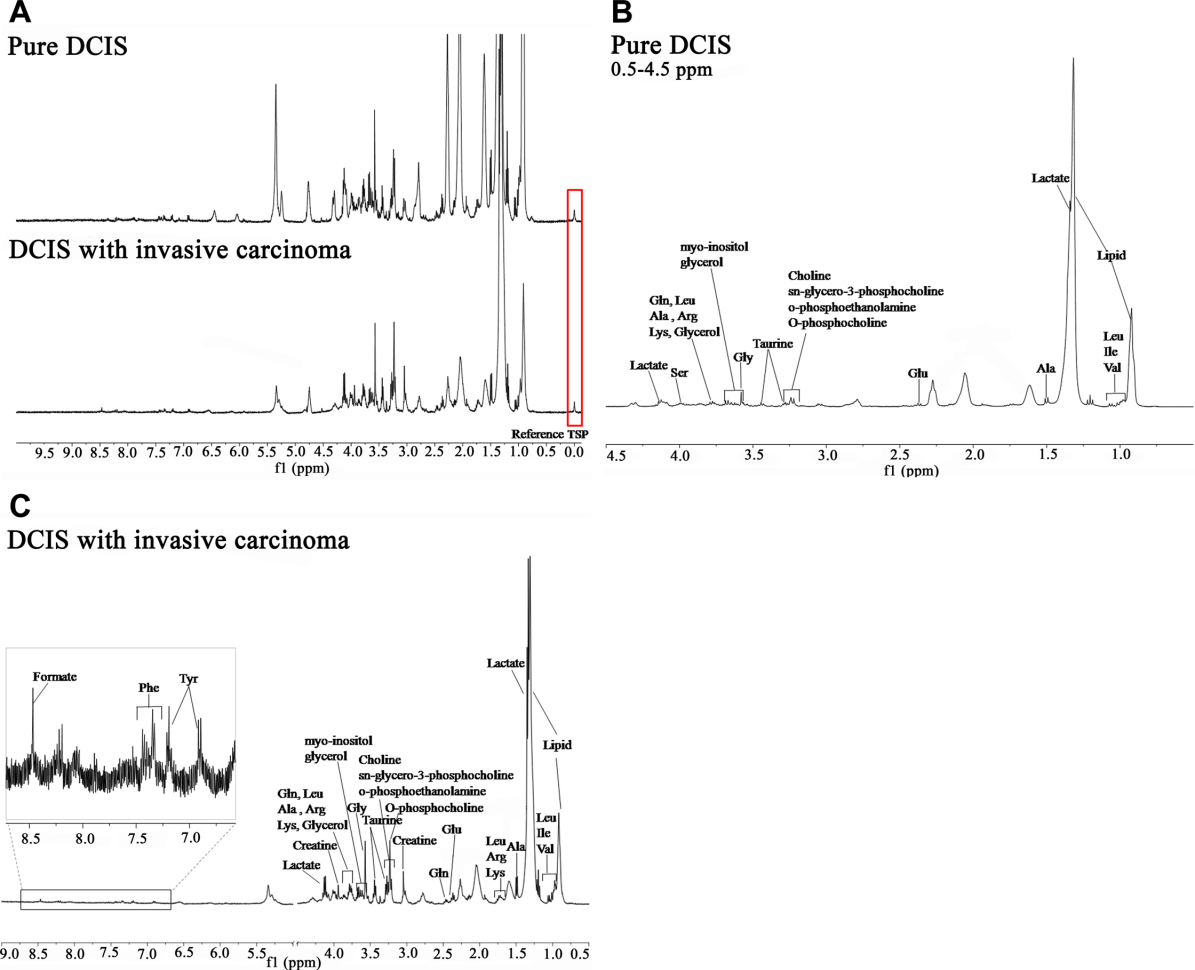
Representative HR-MAS 1H NMR spectra. (a) Representative spectra including TSP (reference material) showing higher metabolite concentrations in pure DCIS than in DCIS accompanying invasive carcinoma. Detailed spectra from pure DCIS (b) and DCIS accompanying invasive carcinoma (c) patients are also shown with metabolite labeling. Because (b) and (c) were based on the largest peak, please refer to Table 2 for the specific values of metabolite concentrations.

In all, HR-MAS 1H NMR spectroscopic data from 60 tumor samples—30 pure DCIS and 30 DCIS accompanying invasive carcinoma—were analyzed (Fig 1). The mean tumor size was 34.2 mm (range, 5–120 mm) for the pure DCIS group. For the DCIS accompanying invasive carcinoma group, the mean size of the invasive component was 10.9 mm (range, 1–36 mm) and the mean size of the intraductal component was 52.7 mm (range, 15–110 mm). The clinicopathologic characteristics of the patients are summarized in Table 1. DCIS accompanying invasive carcinoma group was associated with HER2 positivity and lymph node metastasis. Other clinicopathological variables did not show the significant differences between the two groups.

**Table 1:** Clinicopathologic Characteristics of the 60 Study Patients.

Clinicopathologic characteristics		Pure DCIS	DCIS accompanying invasive carcinoma	p value
**Patient age**	**Years**	54.0	48.7	0.066
**Nuclear grade**	**Low**	21	17	0.284
	**High**	9	13	
**ER status**	**Positive**	24	16	0.054
	**Negative**	6	14	
**PR status**	**Positive**	18	14	0.301
	**Negative**	12	16	
**HER2 status**	**Positive**	11	17	0.031
	**Negative**	13	13	
	**Equivocal**	6	0	
**Molecular subtype**	**Luminal**	24	16	0.054
	**TNBC or HER2**	6	14	
**HER1**	**Positive**	3	1	0.612
	**Negative**	27	29	
**CK5/6**	**Positive**	5	2	0.424
	**Negative**	25	28	
**P53**	**Positive**	11	11	1.000
	**Negative**	19	19	
**LN metastasis**	**Positive**	0	5	0.001
	**Negative**	24	25	
	**N/A**	6	0	

### HR-MAS 1H NMR Spectra and Statistical Analysis

**Fig 2:**
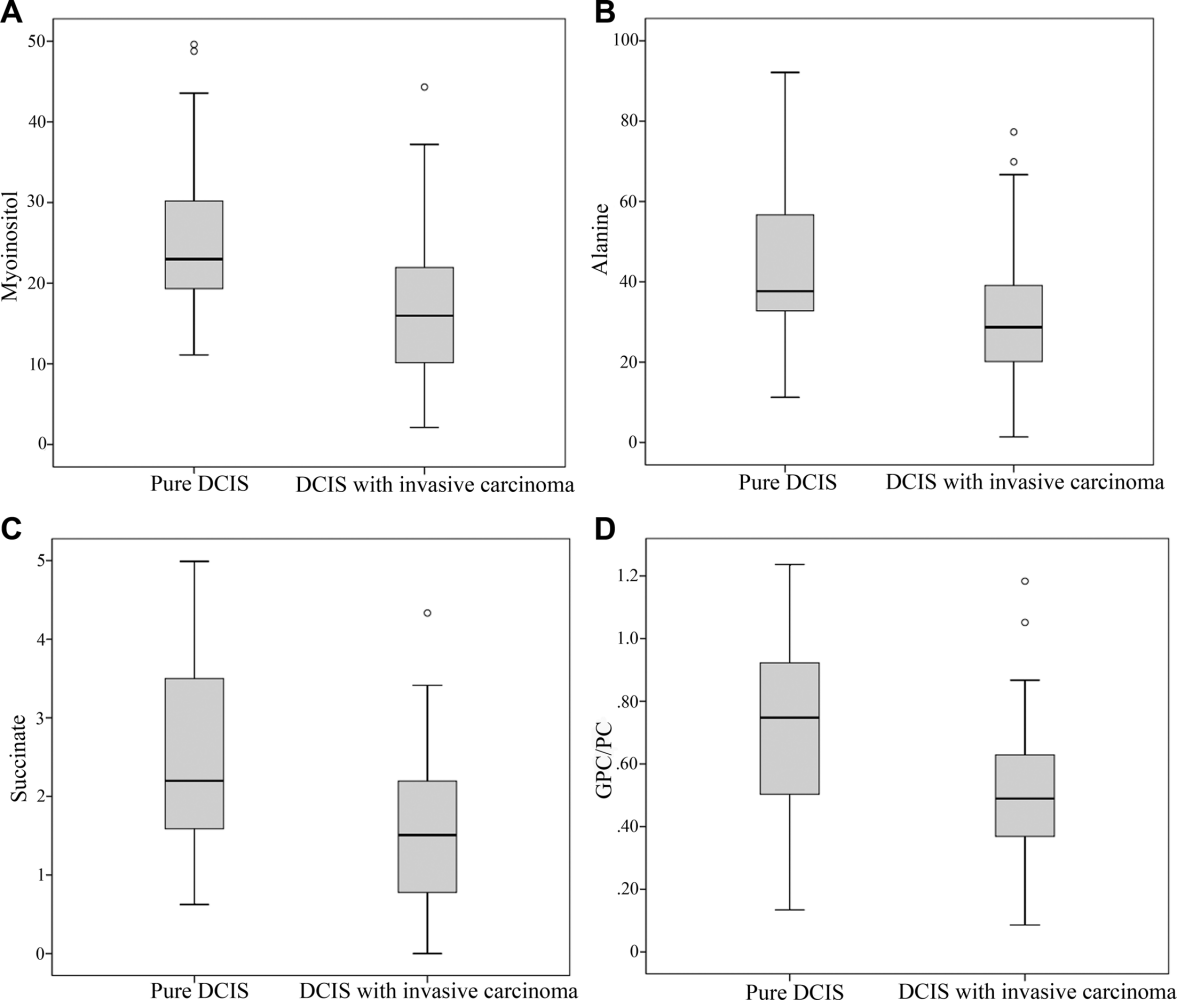
Correlations between the tumor type and metabolite concentrations. Box and whisker plots displaying the correlation between the tumor type and the metabolite concentrations of myo-inositol, alanine, succinate, and the GPC-PC ratio. The boxes represent the median, quartile, and extreme values for the groups.

HR-MAS 1H NMR spectra quantified and identified various metabolites in the 60 tissue samples (Table 3). The mean and median tCho concentrations were 31.52 mM and 26.35 mM, respectively. By univariate analysis (Table 2), the metabolite concentrations of choline-containing compounds did not differ significantly between the pure DCIS and DCIS accompanying invasive carcinoma groups (p = 0.231). However, the concentration of m-Ins (p = 0.001, Bonferroni-corrected p = 0.016), Ala (p = 0.034), and Suc (p = 0.003, Bonferroni-corrected p = 0.048) were significantly higher in the pure DCIS group than in the DCIS accompanying invasive carcinoma group (Fig 2). The concentration of Ala was higher in the pure DCIS group than in the DCIS accompanying invasive carcinoma group (p = 0.034), although the difference did not show statistical significance after Bonferroni correction (p = 0.544). The pure DCIS group showed a higher GPC/PC ratio than the DCIS accompanying invasive carcinoma group (p = 0.004). However, the difference did not reach statistically significance after Bonferroni correction (p = 0.064).

**Table 2:** Comparison of Metabolites Obtained by HR-MAS 1H NMR Spectroscopy Between Pure DCIS and DCIS Accompanying Invasive Carcinoma.

Metabolite or metabolic ratio	Pure DCIS	DCIS accompanying invasive carcinoma	Uncorrected p value	Corrected p value^a^
**Cho**	9.08 (5.20–14.72)	6.93 (5.20–10.14)	0.060	0.960
**PC**	11.20 (6.19–17.48)	10.67 (7.32–15.13)	0.915	NA
**GPC**	7.19 (4.59–11.97)	5.45 (3.67–9.18)	0.087	NA
**tCho**	28.34 (17.61–45.56)	25.18 (15.35–31.75)	0.231	NA
**Cr**	6.94 (4.68–14.40)	5.93 (4.03–11.52)	0.404	NA
**Gly**	47.44 (26.79–87.57)	41.28 (28.74–53.34)	0.187	NA
**Tau**	38.44 (29.99–67.21)	40.01 (25.57–50.07)	0.355	NA
**m-Ins**	22.98 (19.32–31.30)	15.97 (10.85–21.72)	0.001*	0.016*
**Ala**	37.67 (32.77–56.68)	28.69 (20.53–39.06)	0.034*	0.544
**Suc**	2.17 (1.59–3.54)	1.51 (0.82–2.14)	0.003*	0.048*
**Cho/Cr**	1.19 (0.65–1.56)	1.03 (0.62–1.58)	0.585	NA
**PC/Cr**	1.39 (1.07–2.07)	1.63 (1.09–2.41)	0.379	NA
**GPC/Cr**	1.03 (0.72–1.38)	0.86 (0.48–1.29)	0.129	NA
**tCho/Cr**	3.78 (3.05–4.62)	3.66 (2.68–4.81)	0.575	NA
**GPC/PC**	0.75 (0.50–0.92)	0.49 (0.37–0.63)	0.004*	0.064
**GPC/Cho**	0.78 (0.56–1.31)	0.67 (0.50–1.17)	0.534	NA

Data represent the median (interquartile range).

^a^ Bonferroni-corrected p value was calculated as the each p value multiplied by the number of tests (n = 16) and Bonferroni-corrected p values less than 0.05 were considered statistically significant.

* Data indicate p values less than 0.05.

**Table 3:** HR-MAS 1H NMR Spectroscopic Values for the Whole Cohort.

Metabolite concentration (mM)	Mean±SD	Median	Metabolic ratio	Mean±SD	Median
**Cho**	8.82±5.78	8.44	Cho/Cr	1.28±0.88	1.09
**PC**	13.69±9.11	10.87	PC/Cr	1.71±0.93	1.57
**GPC**	9.00±9.72	6.54	GPC/Cr	1.05±0.62	0.98
**tCho**	31.52±22.71	26.35	tCho/Cr	4.04±1.96	3.73
**Cr**	9.47±7.85	6.40	GPC/PC	0.78±0.94	0.61
**Gly**	49.87±31.42	42.57	GPC/Cho	1.41±2.44	0.74
**Tau**	47.11±34.12	39.71			
**m-Ins**	22.72±12.98	20.21			
**Ala**	36.78±20.14	34.62			
**Suc**	2.23±1.77	1.82			

**Fig 3:**
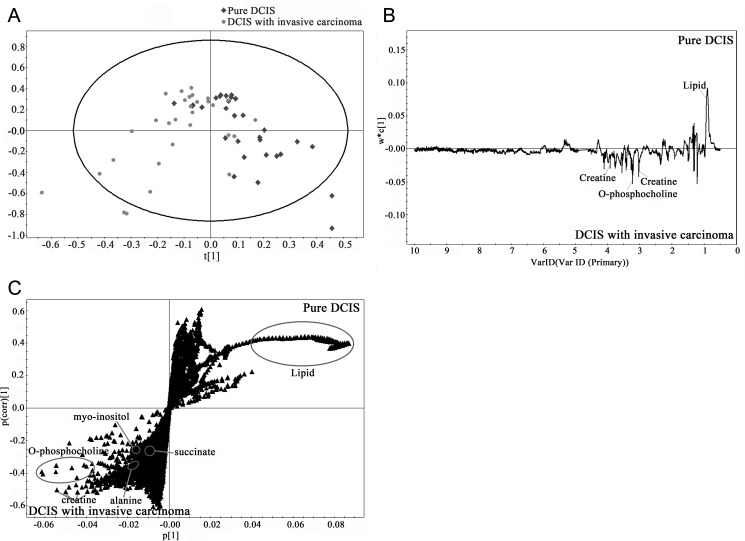
HR-MAS spectra plots according to the tumor type. OPLS-DA score (a), loading (b), and S-plots (c) of the HR-MAS spectra according to the tumor type. The score plot demonstrates a degree of separation between the two groups. The loading and S-plots identify the marker metabolites that are responsible for the separation of the pure DCIS and DCIS accompanying invasive carcinoma groups.

As it was difficult to identify the differences between the pure DCIS and DCIS accompanying invasive carcinoma groups by simple visual inspection due to the large intra-group variation, we performed multivariate statistical analysis for a more holistic view of the data. In the multivariate analysis, OPLS-DA separation models were built with the HR-MAS 1H NMR spectral data according to the tumor type. Overall, the OPLS-DA score plot revealed that the samples showed a degree of separation between the pure DCIS and DCIS accompanying invasive carcinoma groups (Fig 3), although there were some samples that crossed over the reference line. The loading and S-plots revealed the marker metabolites that were responsible for the separation of the pure DCIS and DCIS accompanying invasive carcinoma groups. Compared with the pure DCIS group, the DCIS accompanying invasive carcinoma group showed higher levels of PC and creatine and lower levels of lipid. The OPLS-DA prediction model demonstrated high sensitivity (86.7%) and specificity (80%) for differentiating pure DCIS from DCIS accompanying invasive carcinoma.

## Discussion

In this present study, we evaluated metabolic profiling using HR-MAS 1H NMR spectroscopy in patients with DCIS with or without an invasive component. Our HR-MAS 1H NMR spectroscopy analysis found several metabolite markers (GPC/PC ratio, m-Ins, Ala, and Suc) that differed between pure DCIS and DCIS accompanying invasive carcinoma. We also found that OPLS-DA models using HR-MAS 1H NMR spectral data were able to partially discriminate pure DCIS from DCIS accompanying invasive carcinoma.

Choline-containing compounds are generally regarded as markers of cell signaling, lipid metabolism, and cell membrane integrity [10,11] and their levels are expected to increase in malignant tumors [12]. In our present analyses, the concentrations of choline-containing compounds did not significantly differ between the pure DCIS and DCIS accompanying invasive carcinoma groups. *In vivo* spectroscopy of tumors provides a sum of total choline-containing compounds and cannot determine individual signals, such as GPC and PC, due to resolution limitations. In contrast, *ex vivo* HR-MAS 1H NMR spectroscopy of cell extracts can resolve individual choline metabolites and quantify GPC, PC, and free choline. In our current study, the GPC concentration was lower in the DCIS accompanying invasive carcinoma group than in the pure DCIS group, however, the difference did not reach statistical significance. Interestingly, the GPC/PC ratio was lower in the DCIS accompanying invasive carcinoma group (p = 0.004), although the difference was not significant after Bonferroni correction (Bonferroni corrected p = 0.064). A previous study showed a decreased level of GPC and an increased level of PC in malignant breast tumors [8]. Considering the model of DCIS progression to invasive breast cancer [13], we assume that DCIS accompanying invasive carcinoma is a transitional phase to invasive breast cancer. Thus, the lower GPC/PC ratio of the DCIS accompanying invasive carcinoma group in our present study could be regarded in this context.

A significantly higher concentration of myo-inositol was noted in our pure DCIS group than in the DCIS accompanying invasive carcinoma group. Myo-inositol, an isomer of glucose that is a precursor in the phosphatidylinositol cycle, is a component of cell membranes and is an essential nutrient required by human cells for growth and survival [14]. Elevation of the level of myo-inositol in the pure DCIS group was possibly associated with the chemopreventive effect of myo-inositol [15].

Due to incomplete understanding of DCIS, appropriate management of DCIS patients is still a clinical dilemma. Currently, we cannot predict which DCIS lesion identified on preoperative biopsy will contain an invasive component on definitive surgery. Therefore, patients with DCIS diagnosed on preoperative biopsy undergo treatment without careful consideration of the possibility of invasive carcinoma. Molecular approaches are being considered, although they have so-far only been used to make decisions regarding post-surgery adjuvant treatment [16,17].

DCIS diagnosis is a relatively common finding after core-needle biopsy of the breast [18]. It is clinically important to recognize the likelihood of an underestimation of invasive disease in patients with DCIS detected on preoperative biopsy because the ability to preoperatively identify patients with DCIS who are likely to represent understaged invasive disease would provide important information for treatment planning, such as sentinel lymph node biopsy [18].

Principal component analysis (PCA) and partial least square-discriminate analysis (PLS-DA) have been used in breast cancer metabolomics [7]. OPLS-DA, which was used in this study for multivariate analysis, differs from PLS-DA in that it rotates the score matrix so that the class-orthogonal variation can be separated from the class-predictive one [19]. OPLS-DA can provide easier interpretation of the factors contributing to class differences in the presence of large intra-group variations such as in the MR spectroscopic data of our study. In our present analysis, OPLS-DA models built with HR-MAS MR metabolic profiles could clearly discriminate between pure DCIS and DCIS accompanying invasive carcinoma. The marker metabolites contributing to the separation of the two groups were PC, creatine, and lipid. The DCIS accompanying invasive carcinoma group showed higher levels of PC and creatine and lower levels of lipid than the pure DCIS group. Although these observations were not in complete agreement with the univariate results, our data show that MR metabolomics can assist in the prediction of the invasive component in patients with DCIS.

Our study had some limitations of note. First, in our study, we could not perform an external validation between the test and the training sample because of the limited sample size, which might overfit the results. However, OPLS-DA model uses the internal cross-validation. Although we did not find other validation methods to prevent overfitting in our OPLS-DA model, R^2^ values of a given model may be used to assess its degree of fit to the original data [20]. In our study, R^2^X and R^2^Y values were 0.805 and 0.409, respectively. It has no standard of comparison or critical value for inferring significance, aside from an empirically inferred acceptable value of ≥ 0.4 for a biological model [21]. And, because we included lesions of variable size and the tissue samples for metabolic profiling of HR-MAS 1H NMR spectroscopy were only a part of whole breast tumors, they might not reflect the metabolic alteration of entire tumors because of the intrinsically heterogeneous nature of breast tumors. Next, we included a relatively small number of patients, which may also have affected the results. The significance of our statistical analysis must be considered in the particular context of a preliminary study in which the number of cases presented was still relatively small. Due to the limited sample size also, we did not evaluate many factors that can affect metabolites, such as immunohistochemistry or tumor size, nuclear grade, or mitotic rate. Because histopathologic examination was not performed on the same sample used for 1H NMR spectroscopy, we could not know the exact content within the sample. For the same reason, it was difficult to prove by histology how much the invasive component was present or not within the sample, which was difficult because our hospital was far apart from the NMR spectroscopy experiment laboratory. Therefore, we obtained two mirror samples for both HR-MAS and histopathology by one experienced pathologist in order to reduce this limitation. Moreover, we did not estimate the exact amount of adipose tissue in order to investigate whether the two groups were comparable or not. However, one experienced pathologist tried to check the sample quality and obtained carefully the tissue samples within the tumor boundary. Nevertheless, pure DCIS may be associated with normal breast stroma than invasive breast cancer due to the characteristics of DCIS itself. Previous studies have shown that tissue metabolite concentrations are highly dependent on the tissue composition (e.g., percentage of tumor cells, fat, and connective tissue) We also included DCIS lesions of variable sizes (1–120 mm) while the average weight of the 1H NMR spectroscopy sample was 25 mg. However, we did not analyze the influence of lesion size on the spectroscopic values, and we cannot exclude the possibility that the percentage of DCIS varied between the samples. And, we used pareto scaling for the multivariate analysis, but this may blow up regions of noise.

In addition, the temperature during the scan time in this study was set to 26°C. Although the temperature was determined after careful consideration, previous studies have shown substantial metabolic changes according to temperature, even at low temperatures [22]. Finally, even though the different metabolite profiles separating DCIS and DCIS accompanying invasive carcinoma were identified, we were not able to determine the meaning of all of the metabolite levels. For a detailed characterization and clarification of the significance of the metabolite differences, further studies combined with known prognostic factors, including genetic and proteomic information, will be required. The direct application of this single study to a clinical setting is limited. However, this is still the first study to determine that HR-MAS 1H NMR spectroscopy can detect metabolic differences in DCIS lesions with or without an invasive component.

In conclusion, metabolic profiling by HR-MAS 1H NMR spectroscopy using the OPLS-DA model shows visual discrimination between these two tumor types. HR-MAS 1H NMR metabolomics of breast tissue may help to distinguish between DCIS lesions with and without an invasive component. Further studies with a larger number of patients will be necessary to establish the distinct role of HR-MAS 1H NMR spectroscopy in patients with DCIS.

## Supporting Information

S1 File Sample details and analysis results. (XLS)
